# Non-invasive assessment of HFpEF in mouse models: current gaps and future directions

**DOI:** 10.1186/s12916-022-02546-3

**Published:** 2022-10-14

**Authors:** María Villalba-Orero, Pablo Garcia-Pavia, Enrique Lara-Pezzi

**Affiliations:** 1 Departamento de Medicina y Cirugía Animal, Facultad de Veterinaria, Universidad Complutense de Madrid, Av. Puerta de Hierro, s/n, 28040 Madrid, Spain; 2 Myocardial Pathophysiology Area, Centro Nacional de Investigaciones Cardiovasculares Carlos III, Melchor Fernández Almagro, 3, 28029 Madrid, Spain; 3 Centro de investigación Biomédica en Red Cardiovascular (CIBERCV), Madrid, Spain; 4 Heart Failure and Inherited Cardiac Diseases Unit, Department of Cardiology, Hospital Universitario Puerta de Hierro Majadahonda, IDIPHISA, Madrid, Spain; 5 Universidad Francisco de Vitoria, Madrid, Spain

**Keywords:** Research, Echocardiography, Phenotype, Mouse models, Heart failure, Preserved ejection fraction

## Abstract

**Background:**

Heart failure (HF) with preserved ejection fraction (HFpEF) prevalence is increasing, and large clinical trials have failed to reduce mortality. A major reason for this outcome is the failure to translate results from basic research to the clinics. Evaluation of HFpEF in mouse models requires assessing three major key features defining this complex syndrome: the presence of a preserved left ventricular ejection fraction (LVEF), diastolic dysfunction, and the development of HF. In addition, HFpEF is associated with multiple comorbidities such as systemic arterial hypertension, chronic obstructive pulmonary disease, sleep apnea, diabetes, and obesity; thus, non-cardiac disorders assessment is crucial for a complete phenotype characterization. Non-invasive procedures present unquestionable advantages to maintain animal welfare and enable longitudinal analyses. However, unequivocally determining the presence of HFpEF using these methods remains challenging.

**Main text:**

Transthoracic echocardiography (TTE) represents an invaluable tool in HFpEF diagnosis, allowing evaluation of LVEF, diastolic dysfunction, and lung congestion in mice. Since conventional parameters used to evaluate an abnormal diastole like E/A ratio, isovolumic relaxation time, and E/e′ may pose limitations in mice, including advanced TTE techniques to characterize cardiac motion, including an assessment under stress, will improve diagnosis. Patients with HFpEF also show electrical cardiac remodelling and therefore electrocardiography may add valuable information in mouse models to assess chronotropic incompetence and sinoatrial node dysfunction, which are major contributors to exercise intolerance. To complete the non-invasive diagnosis of HF, low aerobic exercise capacity and fatigue using exercise tests, impaired oxygen exchange using metabolic cages, and determination of blood biomarkers can be determined. Finally, since HFpEF patients commonly present non-cardiac pathological conditions, acquisition of systemic and pulmonary arterial pressures, blood glucose levels, and performing glucose tolerance and insulin resistance tests are required for a complete phenotyping.

**Conclusion:**

Identification of reliable models of HFpEF in mice by using proper diagnosis tools is necessary to translate basic research results to the clinics. Determining the presence of several HFpEF indicators and a higher number of abnormal parameters will lead to more reliable evidence of HFpEF.

## Background

Heart failure (HF) is a major public health problem affecting 26 million people worldwide [1]. About half of HF patients suffer from HF with preserved ejection fraction (HFpEF) [2–4]. HFpEF is a clinical syndrome that develops following a complex interaction of several risk factors that cause organ dysfunction and ultimately show clinical symptoms [5–8]. In contrast to HF with reduced ejection fraction (HFrEF), HFpEF prevalence is increasing and, to date, large clinical trials have failed to reduce cardiovascular mortality in these patients [9–11]. Some factors hampering the development of therapeutic tools for HFpEF include the complexity of this syndrome and the heterogeneity of its population [11], but also failures in the translation of basic research results to the clinic [12–14]. Too often, HFpEF animal models show elevated left ventricular (LV) filling pressures and/or diastolic dysfunction but they seldom demonstrate the development of HF, a *sine qua non* condition to recapitulate human HFpEF [14, 15]. As it happens in humans, diastolic dysfunction may or may not lead to HF, and therefore, basic science reports merely describing cardiac dysfunction should not be published using the term “heart failure,” as results presented will not be translational for HFpEF in human patients, hampering advance in this field. A proper used of the term HFpEF in murine models might be imperative to improve understanding of this complex syndrome. This necessarily requires researchers to meticulously address the model.

Non-invasive HFpEF diagnosis remains challenging in humans [16], even after the identification of several diagnostic criteria, including symptoms such as shortness of breath, fatigue, oedema, tachycardia, and exercise impairment, echocardiography findings related to diastolic dysfunction, morphological changes in the heart, and increased circulating natriuretic peptides [5, 16–18]. In order to standardize clinical HFpEF diagnosis, two main scores have been developed, based on symptoms and several echocardiography findings together with the presence of comorbidities (H2FPEF score) [19] or with increased blood natriuretic peptides (HFA-PEFF score) [16]. However, both scores present discrepancies and 41% of suspected HFpEF patients are only properly classified by one of these scores but not the other one [20]. Therefore, the clinical dilemma in diagnosis remains incompletely solved, warranting further investigation.

**Fig. 1:**
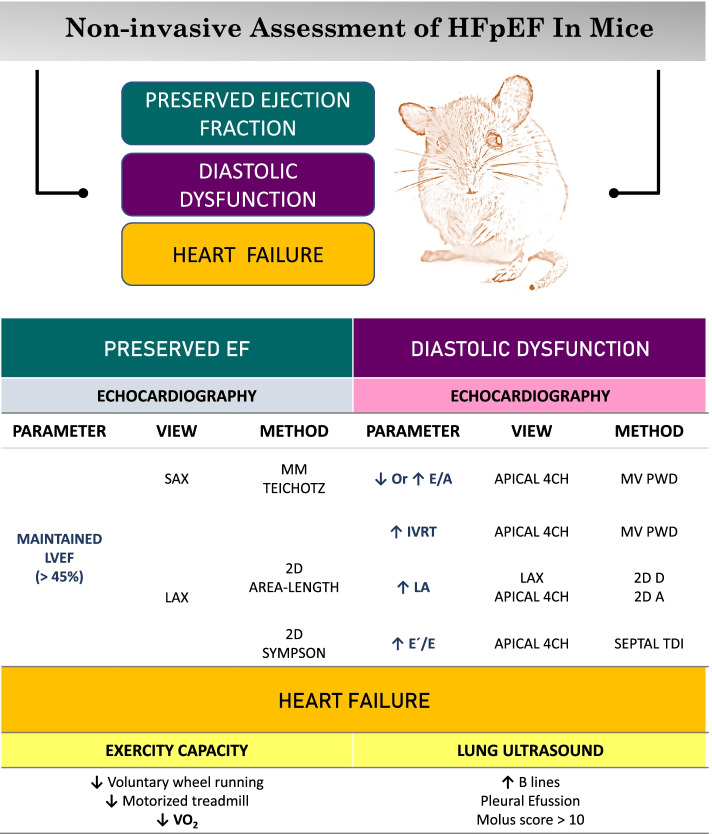
A protocol guide to assess heart failure with preserved ejection fraction in mice. Researchers should look for evidence of three key pathological conditions: preserved ejection fraction, diastolic dysfunction, and heart failure. The most valuable diagnostic tools in mice are described

Mice are the most widely used laboratory animal in translational research and they are also the most popular animal when trying to model human HFpEF [13]. Considering the difficulty in diagnosing HFpEF in clinical patients, who can verbalize symptoms, it is not difficult to envisage that the unequivocal assessment of this syndrome in mice poses several difficulties, ranging from the reliable detection of diastolic dysfunction to lung congestion. Based on the human scores, similar classifications have been recently proposed in mice [12, 13]. Interrogating a mouse model of HFpEF necessarily requires assessing major features defining this complex clinical syndrome. The cornerstone for a precise diagnosis of HFpEF is to highlight the simultaneous presence of a preserved LV ejection fraction (EF), diastolic dysfunction, and HF (Fig. 1).

Non-invasive assessment in mice enables repeated analysis of multiple parameters while causing minimal discomfort to animals. Apart from the evident agreement with the 3Rs posing a more ethical and responsible care and used of animals, it also enables to better demonstration of similarities with the human phenotype, as in persons hardly ever invasive procedures are performed to diagnose cardiac function and HF. For this reason, this work aims to address the key steps in non-invasive diagnosis of HFpEF in mouse models, with a main focus on non-invasive imaging techniques, as they represent a gold standard in the evaluation of HFpEF in human patients. We also highlight gaps and future directions for a global assessment of these mouse models.

## Main text

Transthoracic echocardiography (TTE) and cardiac magnetic resonance (CMR) are the non-invasive imaging techniques most commonly employed for the assessment of HFpEF [16, 21]. Echocardiography is by far the most commonly used in humans and rodents [21]. It is fast, cheap, and safe, and provides valuable information on abnormalities in cardiac structure and function [22]. In contrast, CMR is more expensive and time-consuming than TTE. Thus, it is not routinely used in large series of animals or for longitudinal evaluations [22]. Mice usually are anaesthetized, and, therefore, the impact on cardiac function of the selected drugs must be considered. TTE requires short acquisition times and can be performed under different drugs, including ketamine, barbiturates, tribromoethanol, chloral hydrate, and inhalant agents [23]. In contrast to volatile anaesthesia, the effect of injected drugs cannot be interrupted at will if a mouse shows severe side effects like bradycardia or bradypnea. For this reason, inhalant agents are widely used despite their dose-dependent contribution to cardiovascular depression. For echocardiography, a single intraperitoneal dose of ketamine (100mg/kg) or isoflurane inhalation (3.0% for induction and 1.5% for maintenance) are recommended, since they do not significantly impact cardiac function in mice [24]. Echocardiography can also be acquired in conscious mice; however, it is more challenging to perform and the stress derived from mouse handling can cause non-physiological sympathetic activation leading to increased heart rate and contractility [23, 24]. CMR requires longer anaesthetic time and a steady deeper anaesthetic plane; thus, it is usually performed using volatile agents, which are preferable to combinations of injected drugs [25].

It is important to remark that cardiac function and size are highly dependent on the mouse strain [26]. Considering the variability between strains reported in biomedical research, unique reference values that might fits all strains are not available.

### Assessing LV ejection fraction preservation and diastolic dysfunction

#### Left ventricular ejection fraction evaluation

LVEF is the fraction of blood ejected from the left ventricle (LV) during one heartbeat, expressed as a percentage [27]. LVEF is the parameter most commonly used as surrogate of cardiac systolic function [28]. It is calculated based on the LV volume in systole (ESV) and diastole (EDV) following the formula: LVEF = [(EDV − ESV)/EDV × 100].

**Fig. 2:**
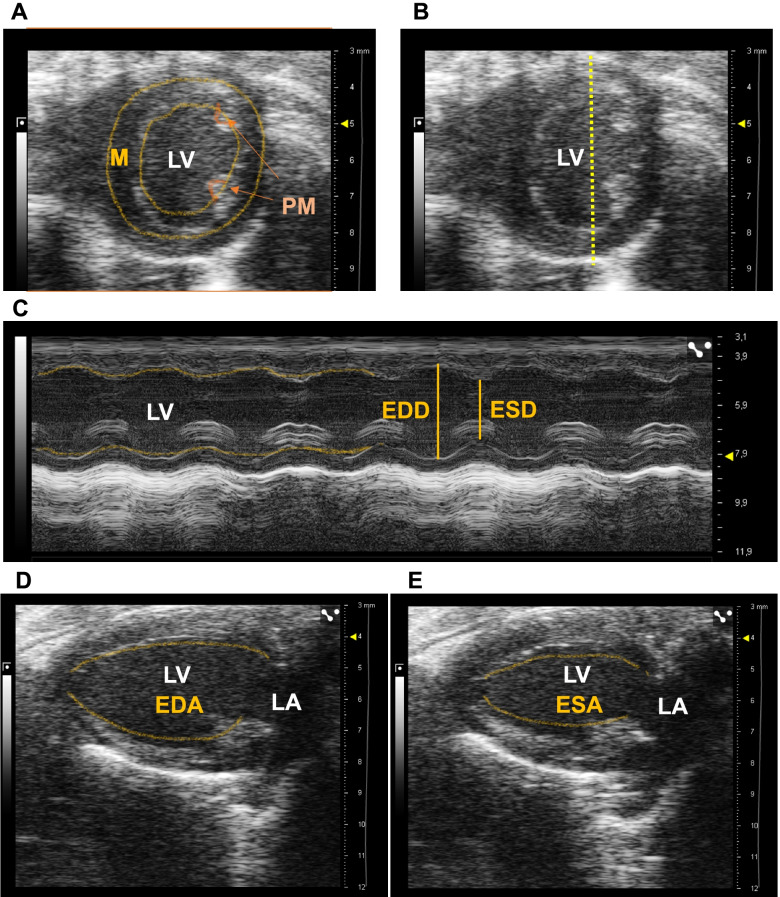
Methods to assess LVEF in mice by echocardiography. **A** Bidimensional short axis (SAX) view of the left ventricle (LV) highlighting visualized structures: myocardial area, LV cavity, and papillary muscles. **B** Line position to measure LVEF in SAX view in M-mode. **C** M-mode of the LV with the end-diastolic and end-systolic diameter measurements in SAX view. In the left part, the endocardial border of the anterior and posterior walls is marked with a yellow dotted line. **D** Bidimensional long axis (LAX) view of the LV at the end of diastole and **E** systole in the same cardiac cycle. The dotted yellow lines mark the endocardial surfaces to obtain the end-diastolic and end-systolic areas. LV, left ventricle; M, myocardial; PM, papillary muscles; EDD, end-diastolic diameter; ESD, end-systolic diameter; EDA, end-diastolic area; ESA, end-systolic area. LA, left atrium

**Fig. 3:**
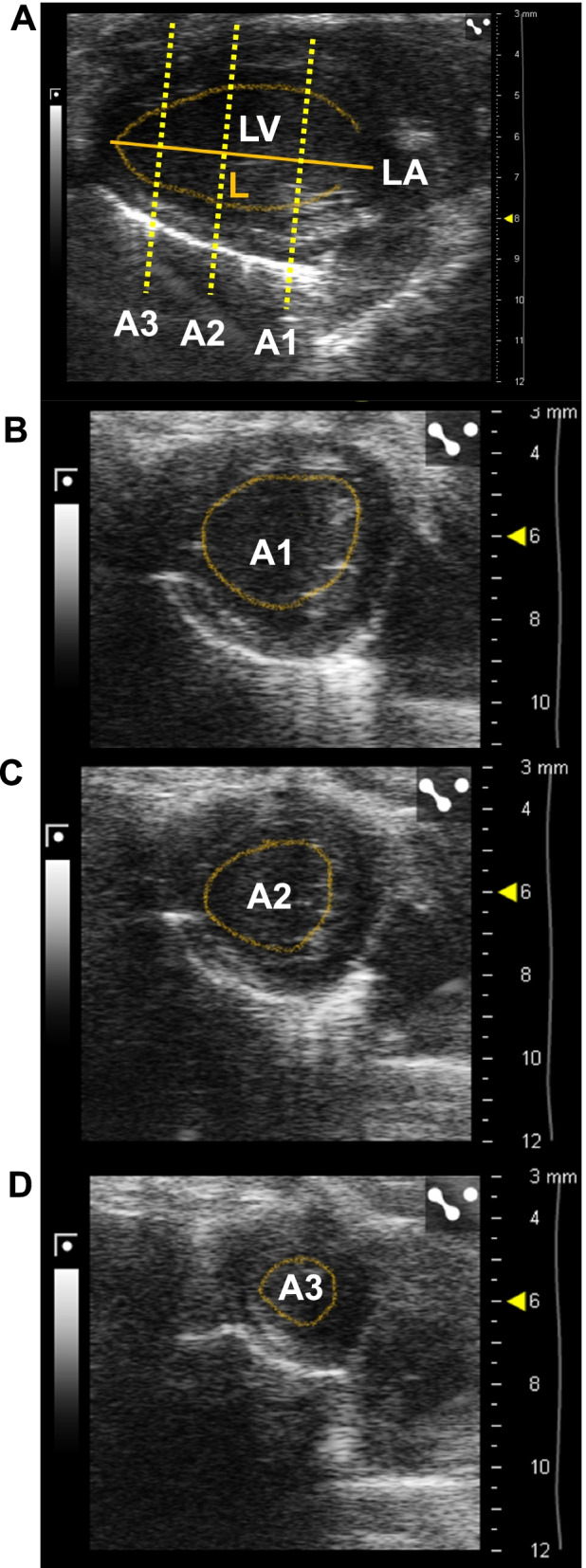
Multiplane rule of discs summation method to assess LVEF in mice. **A** Bidimensional long axis (LAX) view of the left ventricle (LV) at the end of diastole. The solid yellow line marks the end-diastolic length of the LV and the dotted yellow line marks the endocardial border. Three parallel dotted yellow lines mark the anatomic position to obtain a short axis (SAX) view of the LV at the base (A1), papillary muscles (A2), and the apex (A3). **B**, **C** Bidimensional SAX views highlighting the endocardial surface to trace the end-diastolic area at the base (**B**, A1), papillary muscles (**C**, A2), and the apex (**D**, A3). LV, left ventricle; LA, left atrium, L, length; A, area

There are several methods to obtain LV EDV and ESV using TTE. The simplest and fastest method is to calculate the volumes according to Teichholz formula (single plane), acquiring images in a short axis view (SAX) in M-mode (Fig. 2A) and displaying the single line from the anterior to the posterior wall, at the level of the papillary muscles [28] (Fig. 2B). This approach generates the LV internal diameter at the end of diastole and at the end of systole (EDD and ESD, respectively, Fig. 2C). The EDV and ESV are automatically calculated by the ultrasound machine as: EDV = (7 × EDD^3^)/(2.4 + EDD) and ESV = (7 × ESD^3^)/(2.4 + ESD) [27, 29]. The two main limitations when using the Teichholz formula are as follows: (1) estimations are performed by assuming the typical symmetry of the LV and (2) LVEF is only assessed at one level [29, 30]. For a more accurate LVEF measurement other techniques can be performed to obtain more precise LV volumes. First, the LVEF can be obtained by the area-length method (single plane), acquiring images in a bidimensional (2D) long axis view (LAX). The LV area is measured by tracing the endocardial line at the end of diastole and systole (EDA and ESA, respectively) (Fig. 2D, E). The ultrasound machine automatically calculates the LV EDV and ESV applying the following formula: EDV = (0.85 × (EDA^2^)/(3 π ventricular length) and ESV = (0.85 × (ESA^2^)/(3π ventricular length )[29, 30]. Another way to obtain LVEF is by using the multiplane rule of discs summations according to Simpson’s method [29–31]. The LV EDV and ESV are determined from the sum of a pack of measured elliptical discs. The length (L) of the LV at the end of diastole and systole is obtained from the LAX view (LVEDL and LVESL, respectively) and subsequently using a SAX view at three levels to trace the EDA and ESA at each level (base, A1; papillary muscles, A2 and apex, A3) (Fig. 3A–D). LV volumes are calculated using a modified Simpson’s rule as: LVEDV = (A1+A2) × (LVEDL/3) + (A3/2) × (LVEDL/3) + (π/6) × (LVEDL/3) and LVESV = (A1 + A2) × (LVESL/3) + (A3/2) × (LVESL/3) + (π/6) × (LVESL/3) [30]. More sophisticated and complex tools are described to obtain LVEF that allow the highest correlation with CMR measurements. For instance, sequential SAX loops performed every 1 mm along the LV provide close agreement with CMR [22]. Nevertheless, as it happens with CMR, these techniques require longer TTE acquisition and analysis times, which may not be worthwhile in HFpEF research. Finally, three-dimension (3D) echocardiography allows digital volumetric reconstruction from multiple serial 2D images to estimate the total LV volume. Theoretically, 3D TTE appears as the most reliable echocardiography method for the assessment of cardiac chamber volume; however, mouse data using this technique are scarce [30, 32].

CMR provides high accuracy and reproducibility in the calculation of ventricular volumes [33]. To calculate LVEF, Simpson’s rule is applied after obtaining the volumetric quantification from a pile of parallel serial slices which may entirely cover the LV [34]. However, in mice, the small cardiac size together with fast heart and respiratory rates impose substantial challenges for functional evaluation [22, 34] making CMR not commendable especially in murine models of HFpEF.

#### Diastolic dysfunction

Diastolic dysfunction is the fundamental feature of HFpEF, characterized by abnormal LV filling due to an impaired LV relaxation and increased LV stiffness, leading to high LV pressures [11]. In contrast to systolic function, diastolic function is difficult to evaluate noninvasively [35]. However, several changes in the LV filling pattern can be detected by different echocardiography parameters making this technique remarkably valuable in mice. The most useful parameters that may indicate the presence of diastolic dysfunction in mice are abnormal mitral inflow pattern, left atrial (LA) enlargement, and abnormal myocardial velocity [36].

**Fig. 4:**
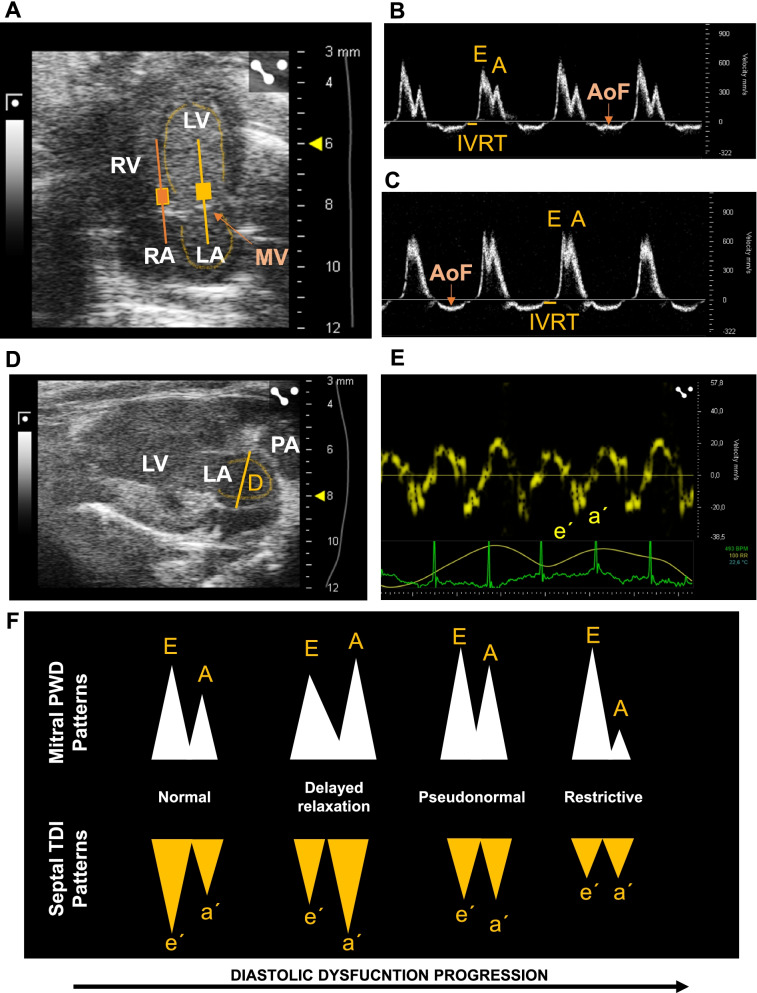
Echocardiography measures used to assess diastolic dysfunction and its progression. **A** Bidimensional apical 4-chamber view highlighting the visualized structures and the sample volume position to obtain a mitral inflow pulse wave Doppler echocardiography (PWD, yellow filled square) and a tissue Doppler image (orange filled square). **B**, **C** Normal (**B**) and abnormal (**C**) mitral inflow PWD showing the early (**E**) and late (**A**) wave peak velocity and the isovolumetric relaxation time (IVRT). **D** Bidimensional LAX view optimized to visualize the left atrial (LA) to obtain a mediolateral end-systolic diameter (**D**). **E** Tissue Doppler imaging at the septal corner of the mitral annulus to obtain early and passive filling myocardial velocities (e′ and a′, respectively). **F** Representative mitral PWD and tissue Doppler waves change as diastolic dysfunction progresses. LV, left ventricle; LA, left atrium, RV, right ventricle; RA; right atrium; MV, mitral valve; E, early wave peak velocity; (A) late wave peak velocity; IVRT, isovolumetric relaxation time; AoF, aortic flow; PA, pulmonary artery; D, diameter; E′, early filling velocity; A′, passive filling velocity; PWD, pulse wave Doppler; TDI, Tissue Doppler imaging

The mitral inflow pattern in mice can be assessed by using an apical 4-chamber view applying pulse wave Doppler echocardiography (PWD) and placing the sample volume in the LV, close to the valve (Fig. 4A) [28]. A transvalvular flow velocity waveflow is provided, and the peak velocity and time intervals can be evaluated [28]. The early (E) wave peak velocity, representing the passive filling, to the late (A) wave peak velocity ratio, representing the active filling due to the atrial contraction (E/A ratio) and the isovolumetric relaxation time (IVRT) are the most robust parameters to evaluate diastolic function in mice [15, 28] (Fig. 4B). Abnormal E/A ratios are well characterized in humans, and different patterns (abnormal relaxation- —grade 1, pseudonormalization—grade 2, restrictive—grade 3) are recognized according to the progression of the diastolic dysfunction [28, 35]. A similar categorization has not been thoroughly described in mice, but similarly to humans, low and high E/A ratios indicate diastolic dysfunction (normal E/A ratio is around 1.5, Fig. 4A–C) [15, 30]. It is important to remark that, occasionally, mice may show fused E and A waves due to their normal high heart rate (450–550 bpm) [28], making it challenging to differentiate a normal from a restrictive pattern. In addition, a pseudonormalized pattern in mice cannot be distinguished from normal mitral inflow pattern by PWD [29]. Evaluating all diastolic parameters, as a whole, helps to overcome confusing mitral inflow patterns.

IVRT is the time interval between the closure of the aortic valve to the onset of filling with the opening of the mitral valve (Fig. 4B, C). It is a measure of myocardial relaxation [37]. To assess IVRT in an apical 4-chamber view, the aortic outflow wave must be visible in the mitral inflow profile [36]. As stiffness increases in the LV, a common feature in diastolic dysfunction, IVRT increases [36, 38]. The main limitation of using IVRT is that it may be shortened or prolonged by high or low heart rates, respectively. Furthermore, IVRT is reduced when there is high atrial pressure [37].

LA size increases as a compensatory mechanism in chronic elevation of LV filling pressure; thus, LA enlargement is a good marker of diastolic dysfunction in humans and animal models [30, 36, 39–41]. Due to the small LA dimensions in mice, accurate measurement using echocardiography may be challenging and it is rarely assessed [36]. However, contrary to mitral valve flow and IVRT, LA size is not sensitive to heart rate and LV loading conditions [41, 42]; therefore, it may provide valuable information in chronic diastolic dysfunction and HFpEF mouse models. Several reports highlight the ability of TTE to assess LA size in mice; it can be measured from a LAX M-mode, LAX 2D mode and apical 4-chamber 2D mode (Fig. 4A, D) [36, 40–42]. The apical 4-chamber 2D mode is suggested as the best view to assess LA area and medio-lateral diameter [30, 41]. A TTE approach has been proposed to obtain the LA volume and assess diastolic dysfunction in mice by using the formula for a prolate ellipse. This requires measuring the antero-posterior, superior-inferior, and medio-lateral diameters [41].

LV myocardial movement and velocities can be assessed using tissue Doppler imaging (TDI) in the apical 4-chamber view in mice by placing the sample volume at the septal corner of the mitral annulus. Two main waves are generated according to myocardial diastolic movement: E′ wave, during early and passive filling and A′ wave, during the active filling. The peak velocity of each wave is then determined (Fig. 4E). Under normal conditions, E and e′ maintain a constant relationship and respond similarly to changes in volume load and pressure gradients in the LV and LA [35]. As diastolic dysfunction progresses, the E/E′ ratio significantly increases. Thus, the E/e′ ratio is one of the parameters that is most widely used to assess diastolic dysfunction in humans [21]. However, in rodents, this parameter is model-dependent [36], and a more accurate characterization is required in HFpEF models to achieve standardization. Figure 4F shows changes in both Doppler image techniques, the inflow mitral pattern, and the tissue Doppler, during diastolic dysfunction progression as it usually happens in humans.

### Cardiac motion characterization using speckle tracking

**Fig. 5:**
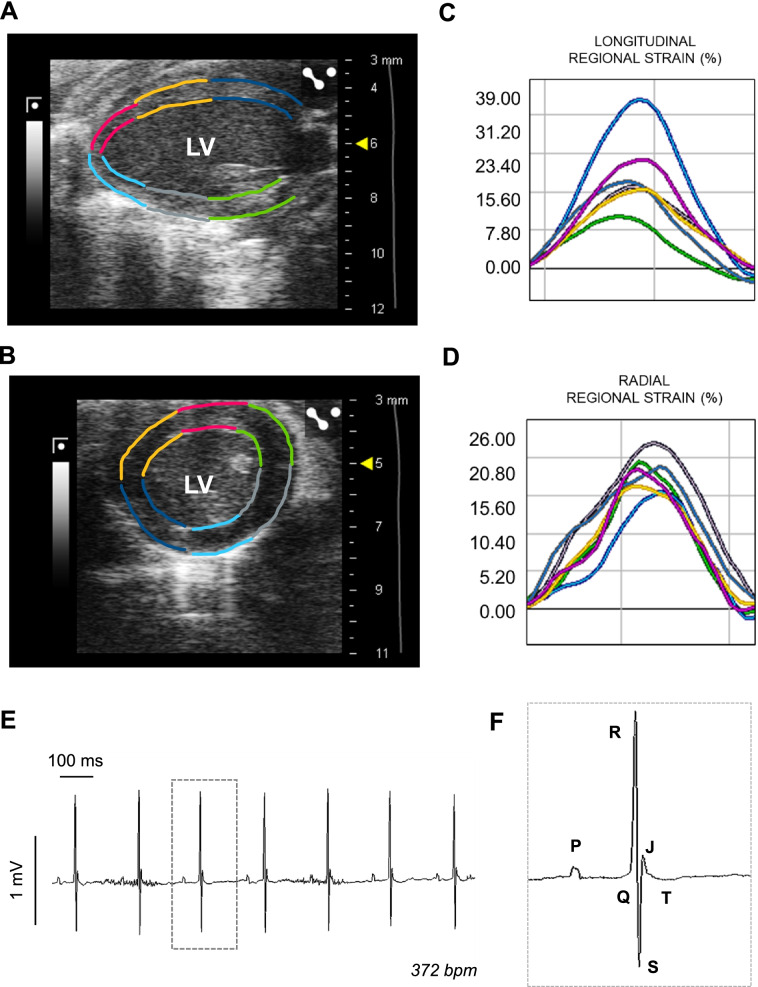
Longitudinal regional strain obtained with speckle tracking echocardiography and mouse electrocardiography. **A** Longitudinal and **B** short axis views of the left ventricle (LV) highlighting the six-region segmentation used for speckle tracking analysis. **C** Longitudinal regional strain of the LV during a single cardiac cycle with each coloured line representing the systolic and diastolic strain of the region with the same colour code as in **A**. **D** Radial regional strain of the LV during a single cardiac cycle with each coloured line representing the systolic and diastolic strain of the region with the same colour code as in **B**. **E** Lead II from a surface electrocardiogram (ECG) obtained from a mouse using a six-lead device. **F** Magnification of the ECG trace included in de dotted square in **E**, highlighting normal ECG waves in mice. LV, left ventricle; bpm, beats per minute

Typically, HFpEF diagnosis is based on the presence of diastolic dysfunction and a preserved LVEF; however, impaired systolic function is also commonly show in human patients with HFpEF, despite a preserved LVEF [43, 44]. Speckle tracing echocardiography (STE) is an emerging technique that enables the evaluation of myocardial function and can be performed also in rodents. The endocardium and epicardium of the LV is traced using a 2D LAX or SAX video loop. The software detects and tracks the speckle pattern of the region traced through the cardiac cycle, which must be visually inspected and manually corrected if necessary. Afterwards, the software segments the LV usually into six regions (basal, mild and apical anterior/posterior; Fig. 5A, B) and provides regional and global (overall mean value) quantification of displacement, velocity, strain (deformation, Fig. 5C, D), and strain rate (rate of change of the deformation over time) [30, 36, 45]. STE can characterize diastolic deformation, which is of major interest for the analysis of HFpEF, using longitudinal strain rate (LSR) values during the early LV filling (reverse LSR). A decrease in this parameter is indicative of abnormal diastolic performance [36, 46]. Main advantages of this tool include the assessment of regional function, dyssynchrony, and its independence from the probe angle [30]. In addition, including reverse LSR solves limitations associated to E/A ratio and the IVRT measurements described above. However, it should be considered that high quality and clear images are required to use this tool, which is not always achievable in mouse echocardiography. Including a global evaluation of potential systolic and diastolic abnormalities in translational research models may improve the understanding of the pathophysiology and the progression of this complex syndrome.

### Stress echocardiography and electrocardiography

Certain abnormalities associated with myocardial mechanical properties described in patients with HFpEF can be assessed under cardiac stress conditions [47]. Stress echocardiography allows an early detection of the syndrome in patients with normal resting echocardiography values or low degree of congestion, as intracardiac pressures may increases only during exercise [48, 49]. In rodents, TTE cannot be performed during exercise, but echocardiography can be acquired under cardiac stress induced by inotropic agents like isoproterenol (1.5–2.0 mg/kg body weight) and may add valuable information to detect early cardiac changes [50, 51].

Electrocardiography (ECG) changes in HFrEF have been widely studied in human patients; however, studies in HFpEF patients are scarce [52, 53]. As in patients with HFrEF, patients with HFpEF present long PR interval, LV hypertrophy morphology, abnormal Q waves, bundle branch blocks, and long QTc [52], although the clinical implication of these findings and the correlation with other phenotypic features were unclear and the diagnostic value of an ECG for these patients remains limited [16]. These limitations may explain the scarcity of ECG phenotyping in preclinical HFpEF research [52]. However, incorporation of routine ECGs in animal models of this syndrome and correlation with other pathological parameters may help to establish the diagnostic value of this technique. To obtain the electrical activity of the mouse heart, a surface human-like 12-lead ECG is performed under light inhaled anaesthesia [54], and lead II is usually analysed. The main differences with human ECG traced are the presence of a J wave at the end of the QRS complex in mice and the lack of an isoelectric ST segment that challenges the definition of the T wave and QT interval (Fig. 5E, F) [55, 56].

Stress ECG represents a valuable tool to highlight pathological features associated with exercise intolerance. In human patients, a poor response of the heart rate to exercise (chronotropic incompetence) and a functional limitation to further sinus node activation during exercise (sinoatrial node dysfunction) would explain at least in part this impaired exercise capability [57, 58]. These features have also been recently observed in preclinical models of HF [57]. To assess the degree of heart rate increase following stimulation in mice, the β-adrenergic agonist isoproterenol is injected intraperitoneally (1–2 mg/kg body weight) during ECG acquisition [56].

### Heart failure diagnosis in rodents

HFpEF diagnosis can be challenging in humans due to non-specific symptoms like fatigue [12], and this is even more difficult in animal which, unlike humans, cannot report symptoms [13]. Typical symptoms in humans presenting HF include fatigue, breathlessness, and exercise intolerance, mainly resulting from fluid accumulation in the lungs and other cavities due to increased venous pressures [59]. Thus, the coexistence of lung congestion represents a useful finding for the clinical diagnosis of HF. These features should also be the basis for HF diagnosis in animals. Domestic animals with HF usually show changes in behaviour, such as refusing to walk or to climb stairs, features that are similar to human symptoms. However, an abnormal conduct is extremely difficult to detect in enclosed mice and could also be indicative of many other diseases and comorbidities. Therefore, researchers must take special care to highlight real evidence of HF in laboratory mice to avoid reporting HFpEF based solely on the presence of diastolic dysfunction.

Non-invasive approaches for HF identification in mice usually encompass some of the following measurements: low aerobic exercise capacity, impaired oxygen exchange, and lung congestion [12, 13, 15, 60]. All of them are used as a surrogate of HF as they can be associated with the most typical symptoms in humans, fatigue and breathlessness.

#### Test to assess exercise intolerance and fatigue

Exercise intolerance is a hallmark of HF [61], and its assessment may be useful in HFpEF models [16]. Mice with pathological cardiac conditions show reduced voluntary as well as forced running capacity [62–65]. Voluntary wheel running is widely used in research. Mice run spontaneously when they have access to running wheels [66], and running parameters (speed, duration and frequency) are recorded every day [63]. Mice should have 1 or 2 days for adaptation before initiating the registration of the running parameters and the running distance peaks after 4 weeks approximately [63, 67]; therefore, this test should be performed during a prolonged period. Voluntary wheel running represents a more natural exercise than forced treadmill and induces less stress on the animal, but researchers do not have total control of the experiment, as mice may choose to run more or less distance voluntarily for a number of reasons [66]. In contrast, motorized treadmill exercise provides well-controlled exercise measurements [68], although it requires negative stimulus (usually an electrical stimulus) and is carried out until exhaustion, increasing stress [66]. Investigators must train mice (usually by electrical shocks) to learn proper running on the treadmill [63]. An important consideration is the lack of standardized procedures for HF diagnosis based on exercise intolerance, similar to the 6 minutes walking or stair climbing tests used in human HF patients [69]. Specific cardiovascular standardized exercise protocols in mice should be develop for HF phenotyping. Either way, the diagnosis of HF merely based on this test might be questionable due to similar limitations to those found in humans [69, 70].

Gas exchange data is compromised by subtle lung congestion during HF leading to exercise intolerance, even if findings are normal at resting conditions. Peak oxygen consumption (VO_2_max) represents the greatest possible amount of oxygen supplied during exercise [63] and is a commonly used measure of exercise capacity in humans [61, 69, 71]. In mice, however, it is a more expensive and technically demanding measurement as mice must exercise in metabolic cages [72]. For this reason, it is reported in a minority of studies [71]. Fifteen minutes running in a metabolic chamber has been suggested as an optimal exercise test to measure this parameter in mice [68, 72].

A main limitation of physical activity and gas exchange tests to assess fatigue in mouse models of HF is that exercise intolerance could arise from pathologies that might be intrinsic or extrinsic to the heart [71], and it does not necessarily imply the development of HF. For instance, exercise tolerance can be decreased in chronic diseases, pulmonary hypertension due to lung disease, ageing, anaemia, obesity or defects in skeletal muscle oxygen extraction [16, 61, 66, 73–75]. These protocols, rather than highlighting fatigue during ordinary gentle physical activity, address physical performance under heavy physical stress. In addition, chronic exercise may improve musculoskeletal and cardiac capacity, complicating the interpretation if the test is performed for a prolonged period [71]. Another important consideration to remark when subjecting mice to exercise is that determining the onset of fatigue may imply operator-dependent variability and introduce a potential bias that should be accounted for. For this reason, acute exercise tests are more appropriate to assess cardiac function than sustained exercise tests [71].

#### Lung ultrasound to assess lung congestion

Lung congestion implies the accumulation of extravascular lung water, caused by increased diastolic filling, and represents a universal mechanism leading to typical symptoms of HF, like dyspnoea and fatigue [12, 15, 76]. The most specific tools to determine pulmonary congestion are those that directly highlight fluid accumulation in the lungs. Lung ultrasound (LUS) has emerged as a valuable method for non-invasive assessment of pulmonary congestion [77]. In addition, LUS has proven its superiority over other diagnostic invasive and non-invasive imaging techniques such as chest radiography or physical examinations [78], and LUS measurements of pulmonary congestion have been associated with traditional clinical markers of congestion in human patients, regardless of LVEF [79].

**Fig. 6:**
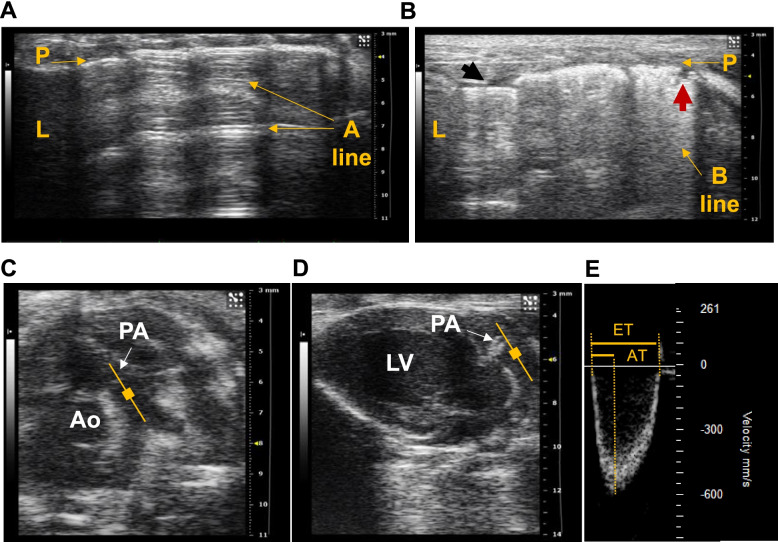
Lung and pulmonary artery evaluation by echocardiography. **A** Normal lung ultrasound (LUS), represented by the presence of A lines. **B** LUS showing broad B-lines indicating pulmonary oedema. The black arrow shows mild pleural effusion and the red arrow shows a disruption in the pleural surface, usually caused by increased hydrostatic pressure in lungs. **C** Short axis view at the level of the greater vessels and **D** a modified angle long axis view used to assess pulmonary artery flow. The yellow filled square indicates the sample volume position to obtain the flow using pulse wave Doppler. **E** Pulmonary artery flow with the ejection and acceleration time represented (ET and AT, respectively). P, pleural line; L, liver. Ao, aorta; PA, pulmonary artery valve

Normal LUS is represented by the presence of A lines, which are horizontal hyperechoic lines and horizontal repetitions artefacts, always equidistant and visible below the pleural line, indicating air (Fig. 6A) [15, 76, 78]. In the presence of extravascular lung water, the ultrasound beam finds subpleural interlobular septa thickened by the oedema and the reflection of the beam creates the so-called B-lines (Fig. 6B). A B-line is a discrete, laser-like, vertical, hyperechoic line, arising from the pleural line that extends to the bottom of the screen [15, 76, 78, 80]. B-lines are presented in patients with cardiogenic lung oedema [78–80], and the number of B-lines increases as New York Heart Association functional classification deteriorates [80]. Pleural effusion is another important marker of pathological fluid retention in lungs [81, 82]. It refers to anechoic fluid leaking into the pleural space, between the lung and the intercostal space and usually close to the liver [15]. The reproducibility of these measurements has encouraged to integrate LUS in clinical practise and should also be considered in animal HF models [15, 78, 83].

To assess pulmonary congestion in mice, left and right-side pulmonary fields can be longitudinally scanned [15, 78]. LUS in mice allows to evaluate sliding during respiration, the profile of predominant lines, echography bedside colour, as well as the presence or absence of Z lines, pleural thickness, pleural defects, and pleural effusion [15]. A score (MoLUS) has been described to assess the presence of HF based of abnormal LUS findings, with a high score indicating the presence of lung congestion [15].

Although pulmonary congestion is associated to HF, it is important to remark that it can also occur in other pulmonary pathological conditions such as non-cardiac pulmonary hypertension and acute respiratory syndrome [16, 75, 84].

#### Serum biomarkers indicating the onset of HF

Circulating biomarkers are widely used in the clinical assessment of HF, as they not only allow to determine the presence of the syndrome, but also provide valuable information of its progression and the effectiveness of a treatment [85].

B-type natriuretic peptides (BNP) and amino-terminal pro-brain natriuretic peptide (NT-proBNP) are both increased in the presence of elevated LV filling pressures, and they are the most widely used biomarkers to assess HF. However, these markers are most useful in acute HF and their clinical value in chronic HF is less clear [86]. In this regard, increased concentration of natriuretic peptides is required in the FFA-PEFF score but is ignored in HF2FPEF [12]. As HFpEF mostly involves a slow progression from diastolic dysfunction to HF, low levels may be found even if the presence of chronic HF, as the heart usually is exposed to lower wall stress. Therefore, caution may be taken when interpreting these biomarkers also in mice [13]. In addition, increased BNP and NT-proBNP indicates a cardiac stress but does not necessarily imply the presence of HF. In rodents, as in humans, natriuretic peptides can be assessed in plasma. Although their expression is reported to be increased in models of HFpEF comorbidities, the presence of lung congestion associated with this increase is not always documented. Therefore, while natriuretic peptides are useful to complete the diagnosis of HFpEF, the mere presence of these biomarkers is not sufficient indicator of the development of this syndrome.

### Measuring common HFpEF comorbidities

Common abnormal conditions shown in HFpEF patients include systemic arterial hypertension (SAH), chronic obstructive pulmonary disease (COPD), sleep apnea, diabetes, and obesity. Ideally, the diagnostic tools used in murine models should be similar to those used in human medicine to get a more accurately translational approach.

In systemic arterial pressure evaluation, since systemic arterial hypertension (SAH) alters the heart’s function and structure, elucidating mechanisms involved in the development of HFpEF necessarily requires monitoring blood arterial pressure. In conscious rodents, the most widely used indirect method for monitoring this parameter is the cuff technique placed in the tail, which determines the cuff pressure at which changes in blood flow occur during occlusion or release of the cuff [87]. Researchers must take into account that using awake animals exacerbates stress and thus, may increase blood pressure. Mice must be trained for at least 5 days before acquiring the final measurements.

In noninvasive assessment of pulmonary arterial hypertension (PAH), echocardiography of the pulmonary artery (PA) dynamics is the main tool for non-invasive assessment of PAH in mice [15, 88]. There are two typical views used for this purpose: a 2D SAX at the great vessels outflow level (Fig. 6C) and a 2D modified LAX view (Fig. 6D). From both, the PA flow can be obtained by displaying the sample region just at the beginning of the PA. The PA acceleration time, PA ejection time, the ratio between the PA acceleration time and PA ejection time (AT/ET; Fig. 6E), PA mean and peak velocity, PA mean and peak gradient, and PA velocity time integral (VTI) are measured. A decrease in AT/ET ratio, velocities, and in VTI indicates the presence of PAH and reinforce a diagnostic of HFpEF if the main features described above are also present.

In the assessment of hyperglycaemia, glucose tolerance, and insulin resistance, accurately performing metabolic tests relies on proper mouse preparation selected careful selection of the protocol. Different fasting and drug administration protocols have been described and researchers must consider pros and cons before choosing the most appropriate one for their model [89].

Measuring glucose levels in mice is simple and fast using a glucometer. The coccygeal vein can be used to obtain a drop of blood after a fasted period [15]. High fasting glucose levels suggest metabolic disorders such as diabetes types 1 and 2. The glucose tolerance test (GTT) is the most widely used test to highlight glucose intolerance [90]. To perform an intraperitoneal, intravenous, or oral GTT, a glucose load (1.5g/kg body weight) is administered to fasted mice (usually following an overnight fast) and blood is collected over a period of time. Plasma glucose and insulin levels are measured at baseline and 15, 30, 60, 90, and 120 min after glucose administration [89]. The results provide a profile of glucose disposal and are a measure of insulin secretion and action. A peak in glucose and insulin plasma concentrations is observed after 10–20 and 60 min, respectively. Increased values or a delay in recovering the basal level indicate glucose intolerance. To determine insulin-resistance, an intraperitoneal insulin sensitivity test (IST) can be used. The protocol is similar to the GTT, except that glucose is measured in response to insulin administration (0.75 UI/kg body weight) [91]. Insulin resistance is diagnosed when the administration of insulin is less effective in decreasing plasma glucose concentration [89, 90].

In multiorgan assessment, another important consideration is the analysis of different organs in the development of HFpEF, which is seldom reported. It is widely recognized that human HFpEF, rather than being characterized by an isolated abnormality in LV diastolic function, is a heterogeneous systemic syndrome characterized by multiple cardiac, vascular, and peripheral pathological features. Accordingly, patients show cumulative risk factors and comorbidities [7, 12, 13, 39, 92]. The pathophysiology of this complex syndrome affects several organs and systems, including lungs, liver, and kidneys, which should not be considered in isolation [8, 11, 93]. Phenotyping mouse models of HFpEF should include the assessment of multiorgan abnormalities and their potential interactions.

## Conclusions

Using proper diagnostic tools in animal models is essential to improve the translation of basic research results to clinical medicine. Highlighting the three key points in HFpEF, preserved ejection fraction, diastolic dysfunction and HF is crucial to evidence a murine model of this syndrome. Since no single parameter can establish the presence of diastolic dysfunction and HF unequivocally, researchers must support the diagnosis by assessing several parameters simultaneously. Finally, to better recapitulate the complexity of this syndrome, a broad evaluation including typical comorbidity conditions associated to HFpEF in humans is crucial.

## Data Availability

Not applicable.

## Abbreviations

A: Late wave peak velocity
CMR: Cardiac magnetic resonance
E: Early wave peak velocity
EF: Ejection fraction
ESA: End-systolic area
EDA: End-diastolic area
ESD: End-systolic diameter
EDD: End-diastolic diameter
ESV: End-systolic volume
EDV: End-diastolic volume
HF: Heart failure
HFpEF: Heart failure with preserved ejection fraction
HFrEF: Heart failure with reduced ejection fraction
IVRT: Isovolumic relaxation time
L: Length
LA: Left atrial
LAX: Long axis view
LVEF: Left ventricular ejection fraction
LVEDL: Left ventricular end-systolic length
LVESL: Left ventricular end-diastolic length
LUS: Lung ultrasound
PWD: Pulse wave Doppler echocardiography
SAX: Short axis view
TDI: Tissue Doppler imaging
TTE: Transthoracic echocardiography
VO_2_max: Peak oxygen consumption
2D: Two-dimension
3D: Three-dimension
